# Coregulation Analysis of Mechanistic Biomarkers in Autosomal Dominant Polycystic Kidney Disease

**DOI:** 10.3390/ijms22136885

**Published:** 2021-06-26

**Authors:** Johannes Leierer, Paul Perco, Benedikt Hofer, Susanne Eder, Alexander Dzien, Julia Kerschbaum, Michael Rudnicki, Gert Mayer

**Affiliations:** 1 Department of Internal Medicine IV (Nephrology and Hypertension), Medical University Innsbruck, Anichstrasse 35, 6020 Innsbruck, Austria; Johannes.Leierer@i-med.ac.at (J.L.); Hoferdikt@gmail.com (B.H.); Susanne.Eder@i-med.ac.at (S.E.); Julia.Kerschbaum@i-med.ac.at (J.K.); Michael.Rudnicki@i-med.ac.at (M.R.); Gert.Mayer@i-med.ac.at (G.M.); 2 Medical Center Hentschelhof, 6020 Innsbruck, Austria; A.Dzien@medicalcenter-innsbruck.at

**Keywords:** autosomal dominant polycystic kidney disease, mechanistic biomarkers, EGFR signaling, angiogenesis

## Abstract

Autosomal dominant polycystic kidney disease (ADPKD) is the most common hereditary kidney disorder leading to deterioration of kidney function and end stage kidney disease (ESKD). A number of molecular processes are dysregulated in ADPKD but the exact mechanism of disease progression is not fully understood. We measured protein biomarkers being linked to ADPKD-associated molecular processes via ELISA in urine and serum in a cohort of ADPKD patients as well as age, gender and eGFR matched CKD patients and healthy controls. ANOVA and *t*-tests were used to determine differences between cohorts. Spearman correlation coefficient analysis was performed to assess coregulation patterns of individual biomarkers and renal function. Urinary epidermal growth factor (EGF) and serum apelin (APLN) levels were significantly downregulated in ADPKD patients. Serum vascular endothelial growth factor alpha (VEGFA) and urinary angiotensinogen (AGT) were significantly upregulated in ADPKD patients as compared with healthy controls. Arginine vasopressin (AVP) was significantly upregulated in ADPKD patients as compared with CKD patients. Serum VEGFA and VIM concentrations were positively correlated and urinary EGF levels were negatively correlated with urinary AGT levels. Urinary EGF and AGT levels were furthermore significantly associated with estimated glomerular filtration rate (eGFR) in ADPKD patients. In summary, altered protein concentrations in body fluids of ADPKD patients were found for the mechanistic markers EGF, APLN, VEGFA, AGT, AVP, and VIM. In particular, the connection between EGF and AGT during progression of ADPKD warrants further investigation.

## 1. Introduction

Autosomal dominant polycystic kidney disease (ADPKD) is the most common genetic renal disorder occurring in approximately one in every 400 to 1000 live births.

The structural hallmarks of this disease are fluid filled, progressively growing epithelial cysts, which can arise from any nephron segment. End-stage kidney disease occurs usually after the fifth decade of life [1,2]. Most cases are caused by mutations in the PKD1 (~78%) or PKD2 (~14%) gene, encoding polycystin 1 (PC1) and polycystin 2 (PC2), but other mutations, some associated with different clinical phenotypes, have also been described [3]. Cysts typically only develop when there is a second (acquired) somatic loss of the normal haplotype [4]. In addition, a “threshold mechanism” has been proposed in a way that PC levels have to fall below a critical level (10 to 30 percent) within a specific tubular epithelial cell in order to trigger the cystogenic process of clonal expansion [5]. 

PC1 and PC2 are expressed in proximal tubules but more pronounced in the distal tubules and collecting ducts [6]. PC1 is involved in the regulation of protein–protein, cell–cell, and cell–matrix interactions and intracellular signaling pathways controlling the regulation of cell proliferation and survival. PC2 is involved in cell calcium signaling. Loss of PC1 or PC2 is associated with low intracellular calcium, increased activity of adenylate cyclase, reduced phosphodiesterase activity and an increase in intracellular cyclic adenosine monophosphate (cAMP). The latter promotes cyst growth by increased CFTR (cystic fibrosis transmembrane conductance regulator)-driven chloride and fluid secretion and by the activation of proliferative pathways. Vasopressin V2 receptor signaling has also been shown to be a potent inducer of cAMP, and in 2015, Tolvaptan, a highly selective vasopressin V2 receptor antagonist, was approved to slow the progression of cyst development and eGFR decline in patients with ADPKD [7] but due to a relatively unfavorable side effect/benefit ratio it has been suggested to limit therapy with tolvaptan to patients at risk of rapidly progressing ADPKD [8]. However, the link from these pathways to disease and the exact mechanism how PC1 and PC2 mutations drive cyst growth remains unclear. Mechanistic molecular biomarkers may inform on dysregulated molecular mechanisms in disease development and/or progression.

The aim of the current study was the identification of protein biomarkers representing different ADPKD-associated pathophysiological mechanisms and the determination of coregulation of these biomarkers in ADPKD patients.

We therefore measured the set of identified mechanistic markers in a cohort of ADPKD patients and a matched cohort of CKD patients as well as in samples from healthy controls. These markers included, among others, growth factors like epidermal growth factor (EGF) or transforming growth factor beta 1 (TGFB1), angiotensinogen (AGT) as member of the renin-angiotensin-aldosterone signaling (RAAS) cascade, vascular endothelial growth factor alpha (VEGFA) and apelin (APLN) being linked to angiogenesis, or vimentin (VIM) being involved in maintaining cell shape and cytoskeletal organization.

## 2. Results

### 2.1. Baseline Characteristics of the Patient Cohorts

We included 37 ADPKD patients in the current study and also analyzed biomarkers in an age-, gender-, and eGFR-matched cohort of 37 CKD patients. The CKD cohort consisted of patients with various diagnoses, such as Hypertensive Nephropathy (*n* = 14), Glomerulonephritis (*n* = 10), Diabetic Kidney Disease (*n* = 3), and CKD of other or unknown origins (*n* = 10) reflecting a broad range of typical causes of CKD. We in addition profiled samples from 10 healthy age- and gender-matched individuals as control group. The mean age was 54 years in the ADPKD and CKD cohort and 47 in the control group. The mean eGFR at time of sample collection was 30.4 mL/min/1.73 m^2^ and 36.1 in the ADPKD and CKD cohort, respectively, and 90.1 in the control group. The use of RAAS inhibitors was comparable between ADPKD patients and CKD patients with slightly more than 50% of patients being on RAAS therapy at time of sample measurements as given in Table 1. The percentage of male and female subjects was also fairly balanced in each of the cohorts.

### 2.2. Biomarker Selection Based on a Generated ADPKD Molecular Model

We collected ADPKD associated molecular features (i.e., genes and proteins) from the OMIM database [9], DrugBank [10], scientific literature, and from a published ADPKD gene expression dataset [11]. The resulting unique set of 1559 molecular ADPKD features was mapped onto a biological network holding protein–protein interactions from three different databases complemented by computationally inferred relations [12].

After graph segmentation analysis with the MCODE algorithm, we identified 25 highly interconnected molecular processes holding in total 528 ADPKD-associated proteins. 285 of these molecular features showed differential regulation in ADPKD on the mRNA level based on the published gene expression signature. Four genes were reported to hold relevant mutations in the OMIM database and 40 proteins represented drug targets of compounds being tested in clinical trials on ADPKD. From the automatic literature search, 88 molecular features were part of the final ADPKD molecular model and 193 molecular features were derived from mechanistic reviews.

Eight proteins with annotation evidence in scientific literature to serve as biomarkers for ADPKD were selected for measurements in the current study. These mechanistic biomarkers covering different molecular mechanisms associated with ADPKD are listed in Table 2.

### 2.3. Biomarker Regulation in ADPKD

**Figure 1:**
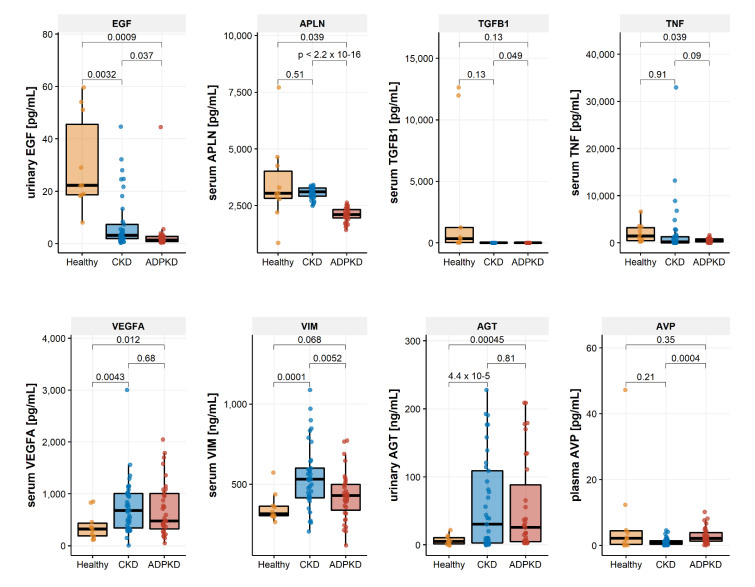
Protein concentrations of the eight investigated molecular biomarkers in the three studied cohorts. *p*-values are based on *t*-tests.

Urinary EGF (*p*-value = 0.001), as well as serum APLN (*p*-value = 0.039) and serum TNF (*p*-value = 0.039) levels were significantly lower in ADPKD patients as compared with the control group of healthy individuals. Urinary EGF (*p* = 0.037) and serum APLN (*p* < 0.001) were also significantly downregulated in samples from ADPKD patients when compared with samples from the group of CKD patients. Serum VEGFA and serum VIM levels as well as urinary AGT levels on the other hand were significantly elevated in ADPKD and/or CKD patients when compared with samples from the healthy control group (Table 3). Plasma AVP was significantly higher in ADPKD when compared with samples from the CKD group. Marker levels in the three cohorts under study are provided in Table 3 along with results from the ANOVA test statistics and graphically displayed in Figure 1.

### 2.4. Biomarker Association with eGFR and Age

**Figure 2:**
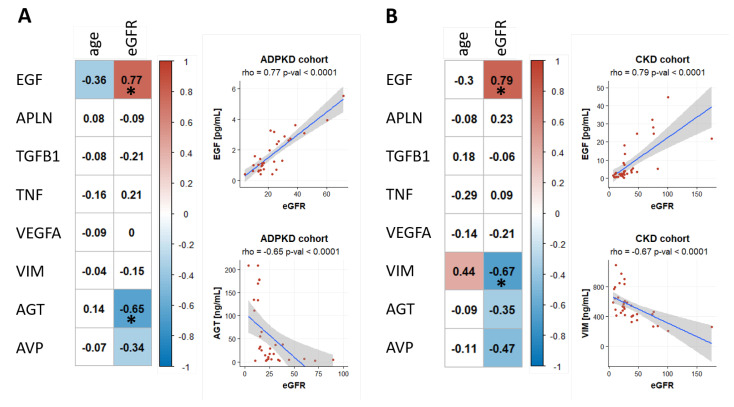
Correlations of biomarker levels with age and eGFR are given when analyzing samples from the ADPKD cohort (panel **A**) as well as samples from the CKD cohort (panel **B**). The Spearman correlation coefficient was used. Significant correlations with *p* < 0.05 are highlighted with colored background. An asterisk (*) indicates correlations also being significant after Bonferroni correction for multiple testing. The EGF outlier with a value of 44.49 was omitted in the scatterplot visualizations for clarity. The value was however included in the correlation analysis.

We evaluated the correlations of biomarker levels with patient age as well as with baseline eGFR values in the ADPKD and the CKD patient cohort. Urinary EGF levels were significantly positively correlated with eGFR values in both cohorts (Spearman rho = 0.77 and 0.79 in the ADPKD cohort and the CKD cohort, respectively; see Figure 2). Urinary AGT was significantly negatively correlated with eGFR in the ADPKD cohort (Spearman rho = −0.65) and also showed a negative correlation with eGFR in the CKD cohort however not reaching statistical significance after correction for multiple testing. Serum VIM levels were negatively correlated with eGFR in the CKD cohort (Spearman rho = −0.67). Plasma AVP was negatively correlated with eGFR in both cohorts with *p*-values above 0.05 after correction for multiple testing though.

We in addition observed a negative trend of urinary EGF values with age in the ADPKD cohort (Spearman rho = −0.36) as well as a positive trend of serum VIM levels with age in the CKD cohort (Spearman rho = 0.44).

### 2.5. Coregulation of Biomakers

**Figure 3:**
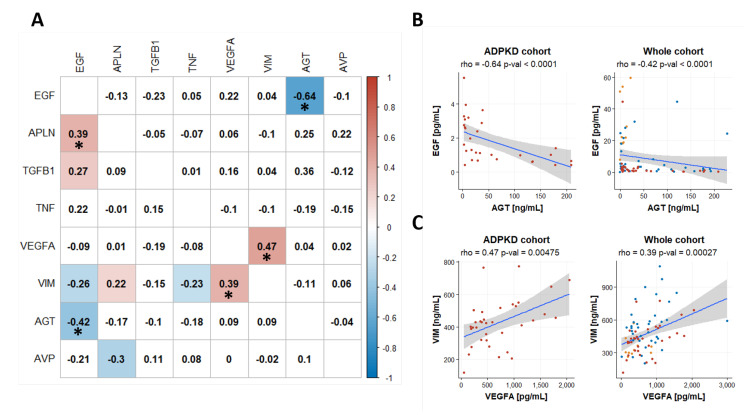
Panel (**A**): Correlogram displaying correlation coefficients of pairwise marker comparisons. The upper half of the matrix indicates correlations based on data from the ADPKD cohort whereas the lower half holds correlations based on data from all three cohorts of the current study. The Spearman correlation coefficient was used in all analyses. Significant correlations with *p* < 0.05 are highlighted with colored background. An asterisk (*) indicates correlations also being significant after Bonferroni correction for multiple testing. Panels (**B**) and (**C**): Scatterplots for urinary EGF and urinary AGT as well as serum VIM and serum VEGFA, respectively. The EGF outlier with a value of 44.49 was omitted in the scatterplot visualizations for clarity. The value was however included in the correlation analysis.

We furthermore determined the pairwise correlations between biomarker levels in the ADPKD cohort but also in the set of all measured samples, i.e., all three cohorts. Urinary EGF levels were significantly negatively correlated with urinary AGT levels in the ADPKD cohort (Spearman rho = −0.64) and also in the full dataset (Spearman rho = −0.42; see Figure 3). Serum VEGFA and serum VIM levels on the other hand were positively correlated in the ADPKD cohort (Spearman rho = 0.47) and also in the full dataset (Spearman rho = 0.39). All pairwise marker correlations are summarized in the correlogram in Figure 3A.

## 3. Discussion

In this study, we analyzed a set of mechanistic protein biomarkers in a cohort of ADPKD patients as well as a cohort of CKD patients and a set of healthy controls. EGF and APLN showed decreased values in ADPKD in urine and serum, respectively with VEGFA, VIM, AGT and AVP levels being increased in ADPKD patients. Urinary EGF was also significantly positively associated with eGFR (rho = 0.77) and urinary AGT was significantly negatively correlated to eGFR (−0.65) in ADPKD patients. Next to a negative correlation of urinary EGF and AGT levels (rho = −0.64), we found a significant positive correlation between serum VEGFA and VIM levels (rho = 0.47) in the ADPKD cohort.

Although the complete pathological mechanisms of ADPKD remain to be elucidated, one of the most evident characteristics is elevated cellular growth and division. Polycystin proteins inhibit cell growth through interactions with several pathways including the mammalian target of rapamycin (mTOR), [13] and Janus kinase (JAK)-signal transducers and activators of transcription (STAT) [14] pathways [15].

Loss of function of the PC1 and/or PC2 proteins leads to ADPKD through multiple signaling pathways and proteins, including the mentioned mTOR, JAK and STAT, but also planar cell polarity (PCP), Wnt, cyclic adenosine monophosphate (cAMP), G-protein coupled receptor (GPCR), cystic fibrosis transmembrane conductance regulator (CFTR), epidermal growth factor receptor (EGFR), mitogen-activated protein kinase (MAPK), cellular Ca^2+^, and the cell cycle [15]. Genetic studies support a threshold model in which cyst formation is triggered by reduced functional polycystin dosage below a critical threshold within individual tubular epithelial cells, due to germline and somatic PKD1 and/or PKD2 mutations, plus mutations of genes (e.g., SEC63, SEC61B, GANAB, PRKCSH, DNAJB11, ALG8, and ALG9) in the endoplasmic reticulum protein biosynthetic pathway, or somatic mosaicism (the presence of two genetically distinct cell populations within one individual resulting from a somatic mutation during embryogenesis) [5]. Proteins investigated in the present study are closely linked to processes being associated with ADPKD progression such as the growth factors EGF and VEGFA, the inflammatory molecules TNF and TGFB1 or apelin as a ligand of a G protein-coupled receptor.

Significantly lower apelin levels were found in the ADPKD cohort of the current study as compared with the CKD cohort. Apelin is an endogenous ligand of the APJ receptor that belongs to the G protein-coupled receptor family [16,17]. In CKD, apelin attenuates renal fibrosis and alleviates renal ischemia/reperfusion injury. The role of apelin in kidney disease in type 2 diabetes mellitus (DKD) is controversial [18]. Serum apelin is higher in diabetes type 2 patients as compared to healthy individuals and it is positively correlated to urinary albumin excretion [19]. Apelin aggravates albuminuria by increasing the permeability of podocytes and glomerular endothelial cells, and podocyte injuries are mediated by apelin triggered ER stress [20]. In a mouse model, increased apelin concentrations in plasma inhibited podocyte autophagy leading to podocyte apoptosis and renal dysfunction in diabetes, thus contributing to the progression of DKD [21]. In ADPKD patients however, apelin levels were found to be lower as compared to healthy controls and lower circulating apelin levels were associated with faster kidney function decline and associated with kidney fibrosis [22]. Apelin was also shown to be an independent predictor of kidney disease progression in ADPKD and patient’s risk for ESKD [23], being in line with the findings of the current study.

A second marker being significantly downregulated in the ADPKD cohort of the current study was EGF. Signaling through EGF receptors (EGFR) is essential for cellular functions like growth, migration, differentiation and proliferation of cells [24]. Dysregulation of EGFR pathway seems to play a role in the pathogenesis of ADPKD [25]. In ADPKD the concentration of EGF in cyst fluid is very low, and EGF plasma concentration as well as urinary excretion are lower in patients with ADPKD than in controls [26,27]. EGF expression was also negatively correlated with age in ADPKD patients which is in-line with previous reports identifying EGF as a renal age-associated gene [28]. EGF expression was previously shown to be downregulated in progressive CKD [29] with lower urinary EGF levels being correlated to intra-renal EGF [30]. Urinary EGF levels were also significantly inversely correlated to urinary AGT levels in the current study. This trend was observable in the ADPKD and the CKD cohort. Urinary angiotensinogen (AGT) is an index of intrarenal renin-angiotensin system (RAS) status [31] with hypertension and progression to CKD being prominent features of (untreated) ADPKD [32]. ADPKD shows significant progression with age when complications due to hypertension are most significant. The activation of the renin-angiotensin-aldosterone system (RAAS) occurs in progressive kidney disease leading to hypertension, which develops before the loss of kidney function and is an important risk factor for progression to ESRD, cardiovascular morbidity and mortality. The RAAS system may also contribute to ADPKD progression by stimulating signaling pathways in the renal cyst cells to promote growth and deregulate epithelial transport [33]. It has been previously shown that AGT levels are increased in ADPKD patients and thus might serve as marker protein [34,35]. The AGT–EGF axis has been subject of excessive research and seems to play an important role in the molecular mechanisms of ADPKD [36,37,38].

We also found significantly elevated plasma AVP levels in the ADPKD cohort as compared with the CKD cohort. Elevated plasma copeptin levels, a surrogate marker for AVP, were found in ADPKD patients [23] and are associated with faster progression [39].

The cysts in ADPKD kidneys contain a well-developed vascular network which is associated with cyst development and fluid secretion into the cysts [40]. The molecular mechanism of neovascularization involves secretion of VEGF, which might be triggered by hypoxia of the tubule cells and of the cysts during their expansion, restricting the process to areas of cyst growth [40]. In addition, tubular hypoxia inducible factor 1α (HIF-1α) is described to have a strong cyst growth-promoting effect in ADPKD mice [41]. Our findings of upregulated VEGFA levels in ADPKD and CKD are in support of these results and might be of special interest, as there are VEGF-, VEGF receptor- but also HIF-1α-antagonists discussed as therapeutic options [42,43].

Tubular epithelial cells of ADPKD cysts express mesenchymal markers like α-smooth muscle actin (α-SMA) and vimentin [44]. Vimentin also represents a VEGF activated target important for VEGF-driven angiogenesis [45]. Increased renal expression of the matricellular protein periostin is accompanied by upregulation of vimentin, which leads to increased mTOR activity, cell proliferation, cyst growth, interstitial fibrosis and acceleration of decline in renal function. This promotes tissue repair pathways leading to faster cyst growth and fibrosis in PKD kidneys [44,46]. mTOR inhibitors have been tested in randomized controlled clinical trials in ADPKD but did not show therapeutic efficacy [47,48]. However, our results support the role of vimentin in ADPKD disease progression as VIM was significantly elevated as compared with the control group with marker levels being highest on average in the CKD group.

Interestingly we could neither see elevated serum levels of TNF nor TGFB1 in the group of ADPKD patients as compared with healthy controls. This is in contrast to previous reports for TNF [49] and TGFB1 [50]. Maybe measurements of these two markers in urine would have been a better choice.

Despite the significant differences and correlations found for the investigated markers, this study has its limitations. First, we were not able to evaluate the prognostic potential of the biomarkers in the ADPKD cohort due to the homogeneous course of progression in the cohort. Second, although we did see significant upregulation of AVP in the ADPKD cohort as compared with the CKD group of patients, in follow-up studies we will probably also include measurements of copeptin, a peptide fragment of AVP [51], that was shown to be an even better biomarker than AVP itself. Another aspect that we could not address in the present study due to the lack of biopsy material from the ADPKD patients is to evaluate the correlations of biomarker levels in body fluids with intra-renal expression patterns. Such a comparison would allow evaluating how good the biomarker levels in body fluids actually represent dysregulations of molecular pathways in renal tissue.

## 4. Methods

### 4.1. Study Design and Populations

For the present study, three age- and sex-matched cohorts were analyzed, namely one cohort of ADPKD patients [*n* = 37], on cohort of CKD patients [*n* = 37], and one control group of healthy individuals [*n* = 10]. The ADPKD and CKD cohorts were also matched for baseline eGFR values. Collected clinical data consisted of age, gender, and eGFR calculated using the MDRD IDMS2 formula (175 × creatinine [mg/dL]^−1.154^ × age [years]^−0.203^ × 0.742 [if female]). We also recorded data on RAAS inhibition at time of sample collection.

Samples were derived from a prospective collection of biomaterials at our institution (biobank), which was approved by the ethics committee of the Medical University Innsbruck (AN4492). Informed consent was obtained from all subjects.

### 4.2. ADPKD Molecular Model Construction

A set of molecular features associated with ADPKD was generated based on information from multiple sources. Molecules associated with ADPKD were identified in OMIM, a database linking genomic alterations to disease phenotypes [9]. Additionally, molecular drug targets of drugs being tested in interventional clinical trials on ADPKD were considered as relevant and added to the input set. Information on drug targets of the investigated drugs was obtained from DrugBank [10]. Differentially expressed genes in human cyst tissues as reported by Malas et al., based on an expression dataset originally published by Song et al., were also taken into account [11,52]. The set of literature-derived molecular features linked via NCBI gene2pubmed associations to publications annotated with the major MeSH term “Polycystic Kidney, Autosomal Dominant” was complemented by molecular features extracted from review articles on the mechanisms of ADPKD [53,54,55,56,57,58].

All extracted molecular features were mapped to the corresponding protein-coding Ensembl GeneID entry. The set of unique Ensembl GeneIDs was used as input set for constructing the ADPKD molecular model, for which the biological hybrid network omicsNET was used. This protein network includes protein–protein interaction data from IntAct, BioGrid, and Reactome complemented with computationally inferred relations [12]. The set of unique molecular features was mapped onto the network and an ADPKD specific induced subgraph was extracted including all molecular features also holding an interaction to at least one other feature of the signature. This condition was true for 1361 protein-coding genes of the total set of 1559 features.

This subgraph was successively forwarded to the Molecular Complex Detection algorithm for identifying highly interconnected clusters of proteins, in the following denoted as molecular process units [59]. The set of identified molecular process units made up the ADPKD molecular model.

### 4.3. Biomarker Panel Selection

Proteins being part of the constructed ADPKD molecular model were evaluated regarding their biomarker evidence based on literature evidence. Biomarker annotation for ADPKD was retrieved from the e.valuation software platform V2.3.4 [60], which uses the MeSH vocabulary as well as information from gene2pubmed associations coupled with text mining procedures to provide biomarker annotation further categorized into the categories prognosis, diagnosis, mechanism and association. Resulting publications were manually screened and markers with evidence in the human setting were selected for measurements in the current study.

### 4.4. Biomarker Measurements

Biochemical analysis of selected protein markers was done in serum, plasma or uncentrifuged urine with commercially available enzyme-linked immuno-sorbent assays (ELISAs). Selection of respective sample material for measuring was based on literature evidence.

Samples were quantitatively measured by ELISA using a calibration curve on each ELISA plate. The amount of immuno-reactive protein was interpolated from optical density (OD) values of calibration curves resulting in concentrations of respective proteins (in pg or ng per mL). For technical normalization, values of blanks (OD of wells without any sample) were subtracted from all other data. Pilot experiments were conducted prior to the actual measurements to determine optimal dilutions for each protein and sample matrix. For the two urinary markers, we also determined the respective marker-to-creatinine ratios and compared these values to the marker concentrations itself. We in the end decided to use the raw marker concentrations as the normalization for creatinine could induce an artificial bias into our dataset the healthy controls showed significantly lower creatinine values. Raw concentrations and creatinine adjusted values of the two urinary markers however showed highly significant correlations.

A listing of used ELISAs with respective sample matrix and dilutions is available in Table 2. Biomarker measurements were performed in duplicates and mean values were used for further analyses. ELISAs not passing quality control defined by determining the detection limit based on signal-to-noise ratio had to be excluded from further analyses.

### 4.5. Statistical Analysis

All analyses were performed in the statistical software R. ANOVA and *t*-tests were used for comparing marker levels between the different cohorts under study. The ggplot2 and corrgram R packages were used for generating boxplots, scatterplots and correlograms, respectively. The Spearman correlation coefficient was used in correlation analysis. Bonferroni correction for multiple testing was applied in the correlation analysis.

## 5. Conclusions

In summary we showed that the mechanistic markers urinary EGF and serum APLN were downregulated in ADPKD whereas serum VEGFA, serum VIM, urinary AGT, and plasma AVP levels were elevated in ADPKD patients. We furthermore found significant correlations between urinary EGF and AGT (rho = −0.64) as well as between VEGFA and VIM (rho = 0.47) in ADPKD patients. Urinary EGF (rho = 0.77) and urinary AGT (rho = −0.65) were in addition significantly correlated with eGFR levels in ADPKD patients. In particular, the EGF-AGT axis warrants further investigation in ADPKD disease progression.

## Figures and Tables

**Table 1:** Key clinical parameters are given for the three cohorts. None of the patients of the ADPKD cohort was treated with tolvaptan. CKD stages according to KDIGO. eGFR = estimated glomerular filtration rate; RAAS = renin angiotensin aldosterone system.

	ADPKD [*n* = 37]	CKD [*n* = 37]	Controls [*n* = 10]
Age (years)			
Min	26	24	26
Max	82	82	62
Mean (SD)	54 ± 13	54 ± 13	47 ± 11
Gender, *n* (%)			
Female	19 (51.4%)	19 (51.4%)	6 (60.0%)
eGFR (mL/min/1.73 m^2^)			
Min	3.8	6.6	71.8
Max	119.5	175.9	129.1
Mean (SD)	30.4 ± 24.9	36.1 ± 32.8	90.1 ± 16.7
CKD stage, *n* (%)			
G1 (≥90 mL/min/1.73 m^2^)	1 (2.7%)	2 (5.4%)	3 (30%)
G2 (60–89 mL/min/1.73 m^2^)	5 (13.5%)	4 (10.8%)	7 (70%)
G3 (30–59 mL/min/1.73 m^2^)	6 (16.2%)	7 (18.9%)	0 (0%)
G4 (15–29 mL/min/1.73 m^2^)	14 (37.8%)	17 (45.9%)	0 (0%)
G5 (<15 mL/min/1.73 m^2^)	11 (29.7%)	7 (18.9%)	0 (0%)
Albuminuria stage, *n* (%)			
A1 (<30 mg/g)	8 (21.6%)	7 (18.9%)	10 (100%)
A2 (30–300 mg/g)	19 (51.4%)	10 (27.0%)	0 (0%)
A3 (>300 mg/g)	6 (16.2%)	15 (40.5%)	0 (0%)
NA	4 (10.8%)	5 (13.5%)	0 (0%)
RAAS inhibitor use, *n* (%)	25 (67.6%)	19 (51.3%)	NA

**Table 2:** Listing of the 8 selected biomarkers along with information on molecular function and pathway assignment. Information on ELISAs used for measurements, sample matrix and dilution is provided in addition.

Symbol	Gene Name	Molecular Function/Pathway Membership	Sample Matrix	Dilution	ELISA (Company/Cat No.)
AGT	angiotensinogen	precursor of angiotensin II; renin angiotensin aldosterone signaling	urine	undil.	Cloud-Clone Corp./SEA797Hu
APLN	apelin	endogenous ligand for the G-protein apelin receptor; angiogenesis	serum	1:5	Cloud-Clone Corp./CED065Hu
AVP	arginine vasopressin	hormonal growth factor; anti-diuretic activity	plasma	undil	Alpco/ 74-VSPHU-E01.1
EGF	epidermal growth factor	growth factor; cell growth, proliferation, and differentiation	urine	1:20	R&D Systems/ DEG00
TGFB1	transforming growth factor beta 1	growth factor; cell growth, proliferation, and differentiation	serum	1:3	Promocell/ PromoKine/ PK-EL-63506
TNF	tumor necrosis factor	proinflammatory cytokine; inflammation, cell differentiation, apoptosis	serum	undil.	Promocell/ PromoKine/ PK-EL-63707
VEGFA	vascular endothelial growth factor A	growth factor; angiogenesis	serum	1:2	R&D Systems/ DVE00
VIM	vimentin	type III filament protein; maintenance of cell shape and integrity of the cytoplasm	serum	1:500	Cusabio Biotech Co.LTD/ CSB-E08982h

**Table 3:** Average marker concentrations plus standard deviations are presented along with the *p*-values of ANOVA test statistics.

	ADPKD [*n* = 37]	CKD [*n* = 37]	Healthy [*n* = 10]	*p*-Value (ANOVA)
EGF [pg/mL]	3.10 (7.66)	7.80 (10.66)	30.20 (17.94)	<0.001
APLN [ng/mL]	2088.18 (298.81)	3074.12 (246.03)	3472.47 (1813.75)	<0.001
TGFB1 [pg/mL]	0.54 (1.42)	0.03 (0.09)	2971.93 (5303.70)	<0.001
TNF [ng/mL]	509.73 (389.43)	2204.97 (5904.43)	2067.02 (2031.68)	0.189
VEGFA [pg/mL]	701.48 (516.17)	753.39 (533.95)	376.85 (267.43)	0.113
VIM [ng/mL]	426.19 (148.68)	546.84 (202.85)	353.27 (90.72)	0.001
AGT [ng/mL]	56.21 (69.50)	60.22 (69.47)	6.58 (7.14)	0.068
AVP [pg/mL]	2.85 (2.41)	1.16 (1.09)	7.34 (14.48)	0.005

## Data Availability

Not Applicable.
